# Superiority of p‐tau217 among plasma biomarkers in diagnostic accuracy in non‐demented persons from the Geneva Memory Center cohort

**DOI:** 10.1002/alz.088800

**Published:** 2025-01-09

**Authors:** Augusto J. Mendes, Federica Ribaldi, Aurelien Lathuiliere, Nicholas J. Ashton, Shorena Janelidze, Henrik Zetterberg, Max Scheffler, Frederic Assal, Valentina Garibotto, Kaj Blennow, Oskar Hansson, Giovanni B. Frisoni

**Affiliations:** ^1^ Laboratory of Neuroimaging of Aging (LANVIE), University of Geneva, Geneva Switzerland; ^2^ Geneva Memory Center, Department of Rehabilitation and Geriatrics, Geneva University Hospitals, Geneva Switzerland; ^3^ Department of Psychiatry and Neurochemistry, Institute of Neuroscience and Physiology, The Sahlgrenska Academy, University of Gothenburg, Mölndal, Gothenburg Sweden; ^4^ Clinical Memory Research Unit, Lund University, Lund Sweden; ^5^ Institute of Neuroscience and Physiology, Sahlgrenska Academy at the University of Gothenburg, Mölndal, Gothenburg Sweden; ^6^ Division of Radiology, Geneva University Hospitals, Geneva Switzerland; ^7^ Geneva University Hospitals, Geneva Switzerland; ^8^ Division of Nuclear Medicine, Geneva University Hospitals, Geneva Switzerland; ^9^ Institute of Neuroscience and Physiology, Sahlgrenska Academy at the University of Gothenburg, Göteborg Sweden; ^10^ Clinical Memory Research Unit, Department of Clinical Sciences, Lund University, Lund Sweden; ^11^ University of Geneva, Geneva Switzerland

## Abstract

**Background:**

The identification of Alzheimer's disease (AD) pathology at an early stage utilizing plasma biomarkers has attracted significant interest due to its potential for improving global screening programs. Different forms of p‐tau measured in plasma, mainly p‐tau217, have demonstrated similar diagnostic accuracy when compared to traditional biomarkers. Moreover, plasma biomarkers associated with neuroinflammation and neurodegeneration, namely GFAP and NfL, respectively, have been found to be elevated in patients with amyloidosis and tau accumulation, even though these conditions are not exclusive to AD.

**Method:**

Our goal was to see if there were any differences in plasma biomarkers between amyloid (A+) and tau (T+) positive groups based on PET scans and compare their diagnostic accuracy in a Geneva Memory Center non‐demented group. Plasma, amyloid‐PET, and tau‐PET were assessed in a total of 100 subjects (CU = 33; MCI = 67). Plasma biomarkers included were p‐tau217, p‐tau231, p‐tau181, GFAP, and NfL. The differences in each plasma biomarker among A+/T+ groups were tested using Kruskal‐Wallis tests. Additionally, we calculated the receiver operating characteristic (ROC) and underlying area under the curve (AUC) in A+ and T+.

**Result:**

All plasma biomarkers revealed significant differences between A‐/T‐ and A+/T+ (p < 0.05), where p‐tau217 showed a higher effect size (δ = 0.98) in comparison with other biomarkers (δ_range_ = 0.31 ‐ 0.67). Moreover, p‐tau217 was the only biomarker displaying significant differences between A‐/T‐ and A+/T‐ (p < 0.001; δ = 0.89). Lastly, p‐tau217 showed a higher AUC for detecting amyloid and tau (AUC_average_ = 0.96) than other p‐tau forms (AUC_average_ = 0.77) and GFAP/NfL (AUC_average_ = 0.69).

**Conclusion:**

Plasma p‐tau217 revealed superior performance in AD pathology detection when compared to other p‐tau forms and plasma biomarkers of neuroinflammation/neurodegeneration. Our results suggest the potential of p‐tau217 to identify AD pathology in a non‐demented population.

